# Sulfated Galactan from *Palisada flagellifera* Inhibits Toxic Effects of *Lachesis muta* Snake Venom

**DOI:** 10.3390/md13063761

**Published:** 2015-06-11

**Authors:** Ana Cláudia Rodrigues da Silva, Luciana Garcia Ferreira, Maria Eugênia Rabello Duarte, Miguel Daniel Noseda, Eladio Flores Sanchez, André Lopes Fuly

**Affiliations:** 1 Department of Molecular and Cellular Biology, Federal Fluminense University, CEP 24020-141 Niterói, Rio de Janeiro, Brazil; E-Mail: anacrs1@yahoo.com.br; 2 Department of Biochemistry and Molecular Biology, Federal University of Paraná, CEP 81531-980 Curitiba, Paraná, Brazil; E-Mails: lugarciaferreira@gmail.com (L.G.F.); nosedaeu@ufpr.br (M.D.N.); 3 Laboratory of Biochemistry of Proteins from Animal Venoms, Research and Development Center, Ezequiel Dias Foundation, CEP 30510-010 Belo Horizonte, Minas Gerais, Brazil; E-Mail: eladiooswaldo@gmail.com

**Keywords:** sulfated galactan, *Palisada flagellifera*, *Lachesis muta*, snake venom, neutralization

## Abstract

In Brazil, snakebites are a public health problem and accidents caused by *Lachesis muta* have the highest mortality index. Envenomation by *L. muta* is characterized by systemic (hypotension, bleeding and renal failure) and local effects (necrosis, pain and edema). The treatment to reverse the evolution of all the toxic effects is performed by injection of antivenom. However, such therapy does not effectively neutralize tissue damage or any other local effect, since in most cases victims delay seeking appropriate medical care. In this way, alternative therapies are in demand, and molecules from natural sources have been exhaustively tested. In this paper, we analyzed the inhibitory effect of a sulfated galactan obtained from the red seaweed *Palisada flagellifera* against some toxic activities of *L. muta* venom. Incubation of sulfated galactan with venom resulted in inhibition of hemolysis, coagulation, proteolysis, edema and hemorrhage. Neutralization of hemorrhage was also observed when the galactan was administered after or before the venom injection; thus mimicking a real *in vivo* situation. Moreover, the galactan blocked the edema caused by a phospholipase A_2_ isolated from the same venom. Therefore, the galactan from *P. flagellifera* may represent a promising tool to treat envenomation by *L. muta* as a coadjuvant for the conventional antivenom.

## 1. Introduction

Envenomation by snakes represents a public health problem especially in the rural areas of Africa, Asia, Latin America and Oceania, in which frequently affects young and economically active people [1,2]. According to the World Health Organization, snakebite is considered a neglected disease [3,4] that affects 20,000 victims per year, with 0.5% of deaths and 8% of morbidity. However these numbers are not exact since snakebites often occur where there is no health unit or hospital to handle notification. Venoms produced by Viperidae snakes consist of a complex mixture of proteins including metalloproteinases (SVMPs), serine proteinases (SVSPs), phospholipases A_2_ (PLA_2_), disintegrins, C-type lectins related proteins, myotoxins, and others [5], that induce local effects (pain, edema, necrosis, inflammation, hemorrhage) and systemic effects (coagulation disturbs, renal and cardiac failure, hemorrhage and neurotoxicity) into victims [6]. Bushmaster species is the largest venomous pit viper inhabiting the Amazon region of several countries of South America and its severe envenomation is characterized by bleeding, renal failure, shock, pain, local hemorrhage, diarrhea, bradycardia, drastic hypotension, and tissue necrosis [7,8]. In the Amazon region of Brazil, the statistical frequency of snakebite caused by *Lachesis muta* is 17% and *Bothrops* 76% and should be considered medical emergency regardless the size of the snake and the venom yield (168–552 mg/snake) [9]. The proteomic characterization of bushmasters (*L. muta* and *L. stenophris*) venom [10] shows that the venom of *L. muta* contains around 30–40 proteins belonging to only 8 toxin families, especially high levels of metalloproteinases of P-I and P-III classes [11]; serine proteases with coagulant [12], plasminogen activation [13], and kallikrein-like [14] activities; phospholipases A_2_ [15,16], among other components which play a key role in bushmaster envenoming.

Snakebite has received little attention from the pharmaceutical industry, governments or academia to improve antivenom therapy. Administration of antivenom is the only effective and accepted therapy for snakebites. However, it has some disadvantages, especially the poor inhibition of local effects [1,4], side effects (fever and/or anaphylactic reactions) and high production cost [4,17]. For this reason, it is necessary to search for alternative neutralizing molecules capable of acting efficiently against the local effects promoted by the snake venoms.

Marine organisms produce molecules with a great chemical diversity derived from primary (lipids, polypeptides and proteins, enzymes and polysaccharides) and secondary metabolism (terpenes, alkaloids and sterols), making them powerful tools for biotechnological use because of their variety of pharmacological and ecological functions [18]. Moreover, seaweeds are known as producers of different polysaccharides, such as galactans, fucoidans, rhamnans, xylans, xylogalactans and xylomannans, with a wide spread of pharmacological effects [19,20,21,22,23]. Despite the various biological activities attributed to seaweed sulfated polysaccharides, only few studies describe these natural compounds as antivenom agents. A sulfated fucoidan from *Fucus vesiculosus* presented a protective effect against the cytotoxic and myotoxic activities of a group of phospholipase A_2_ myotoxin from crotaline snake venoms [24]. When compared to high-molecular weight fucoidan obtained from *F. vesiculosus*, fragments of this sulfated polysaccharide obtained after partial hydrolysis presented lower capacity to prevent muscle necrosis in mouse after injection of purified myotoxin or crude venom [25]. These results were contradictory with the author expectations, which hypothesized that smaller fucoidan fragments, being of higher diffusibility to tissues, may present a higher antivenon activity [25].

Several biological activities have been attributed for galactans obtained from seaweeds, including antiviral, anticoagulant, antiangiogenic and antitumor effects [26,27,28,29]. However, as far as we know, there are no studies reporting the antivenom activity of these natural polymers. Sulfated galactans are the main polysaccharides produced by red seaweeds and present a basic structure constituted by repeating units of 3-linked β-d-galactose and 4-linked α-galactose, named A and B units, respectively [26]. Frequently the B units are found as 3,6-anhydro-α-galactose. Additionally, the B units with enantiomeric configuration l (α-l-galactose or 3,6-anhydro-α-l-galactose) characterize a galactan belonging to agaran group, whereas the B units with enantiomeric configuration d (α-d-galactose or 3,6-anhydro-α-d-galactose) characterize a galactan belonging to carrageenan group. In this work, we have used a purified sulfated galactan from *Palisada flagellifera* named FHS-3. The structures of the sulfated polysaccharides (xylomannans and galactans) produced by this species were previously characterized and described with details [30,31]. Regarding its galactans, *P. flagellifera* biosynthesizes a family of highly complex sulfated, methylated and pyruvylated agarans [31].

In this paper, we evaluate the ability of the sulfated galactans isolated from the red seaweed *P. flagellifera* to neutralize some toxic activities of *L. muta* venom. Moreover, its neutralizing ability was also examined against a PLA_2_ isoform isolated from the same venom (named LM-PLA_2_-I), suggesting the potential use of these natural polymers in the treatment of snakebite accidents.

## 2. Results and Discussion

Accidents by *Lachesis* venom should be considered a life-threatening disease. Antivenom therapy is effective in neutralizing the systemic toxic effects, if administered in time (within 60 min of the accident), but it is ineffective or less effective against local tissue destruction at the bite site [32]. So, in addition to mortality, victims survive with permanent physical sequelae due to local tissue necrosis. Thus, there is a great demand for new molecules from natural sources with antivenom effects. Crude extracts of plants or their isolated molecules with antivenom property have been reported [33,34], but little investigation has been performed with seaweeds [35] and, even less with the polysaccharides from such source [24,25]. Anticoagulant and antiviral effects of sulfated polysaccharides from seaweeds are well established and, in some cases a possible correlation between chemical structure and mechanism of action has been proposed [36,37,38,39,40]. Furthermore, some reports describe the neutralizing capacity displayed by sulfated fucoidans from brown seaweeds against snake venoms [25]. So, here we report the inhibitory effect of a high complex sulfated, pyruvylated and naturally methylated agaran type galactan (named FHS-3) from the red seaweed *P. flagellifera* against some *in vitro* and *in vivo* toxic activities of *L. muta* venom. FHS-3 reduced hemolytic, proteolytic, coagulation, hemorrhagic and edematogenic activities.

### 2.1. Neutralization of Coagulation

**Figure 1:**
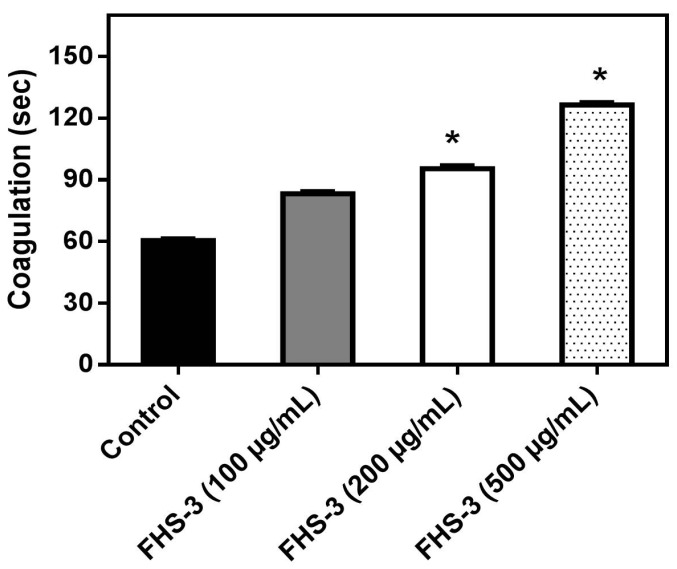
*L. muta* venom (10 μg/mL) was incubated with FHS-3 at 100, 200 or 500 μg/mL for 30 min at 25 °C, then the mixture was added to plasma and clotting time was recorded, as described in the Methods. Data are expressed as means SEM of three individual experiments (*n* = 3). * Significance level (*p* < 0.05) when compared to control (venom mixed with saline).

Abnormal blood clotting and/or bleeding are frequently observed in snake bites and are of clinical relevance [41]. *L. muta* venom (10 μg/mL) clotted plasma in *ca.* 60 s and such venom concentration, that represents one MCD, was incubated with FHS-3 at different concentrations (100, 200 or 500 μg/mL). Figure 1 shows that FHS-3 inhibited *L. muta*-induced coagulation with a dose-dependent profile. FHS-3 did not induce coagulation.

### 2.2. Neutralization of Hemolysis and Proteolysis

**Figure 2:**
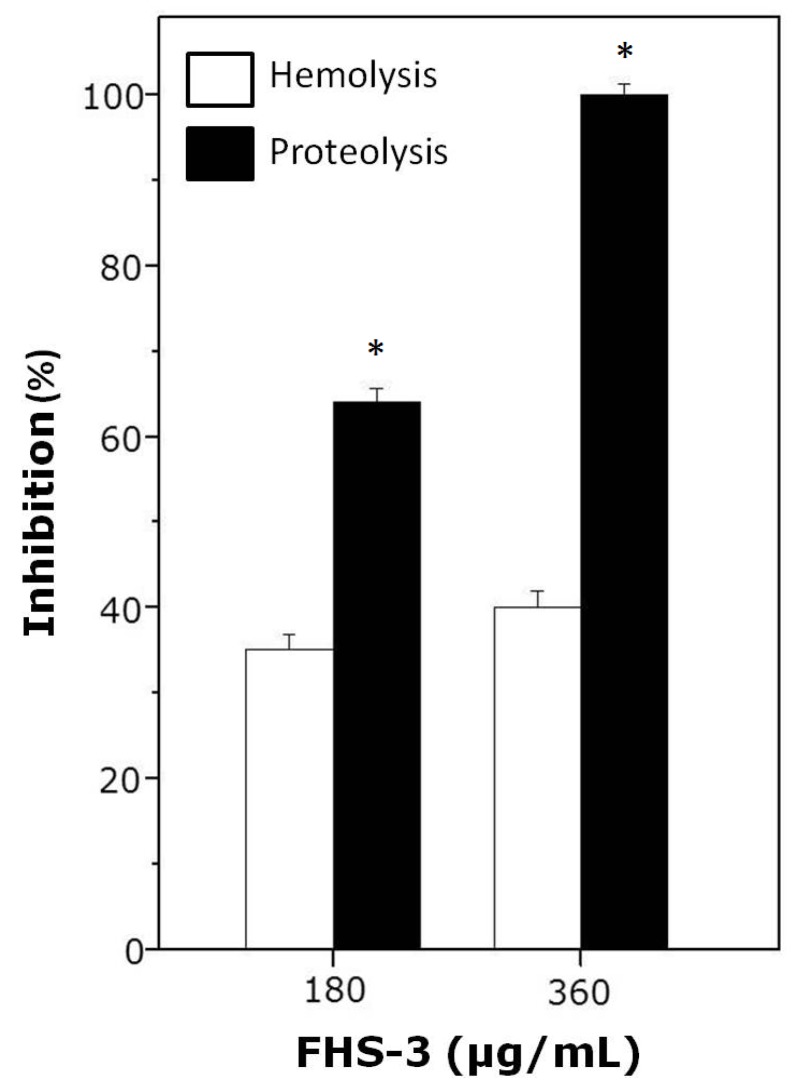
*L. muta* venom (18 μg/mL) was incubated with FHS-3 (180 or 360 μg/mL) for 30 min, and then hemolysis or proteolysis was determined. Data are expressed as means SEM of three individual experiments (*n* = 3). * Significance level (*p* < 0.05) when compared to white columns.

As shown in Figure 2, FHS-3, at 1:10 (180 μg/mL) or 1:20 (360 μg/mL) venom:polysaccharide ratio (w/w) inhibited hemolysis and proteolysis induced by *L. muta* (18 μg/mL). As seen, FHS-3 inhibited more efficiently the proteolytic than the hemolytic activity of *L. muta* venom, since at 1:20 ratio, FHS-3 inhibited 100% of proteolysis and only 40% of hemolysis, maybe because *L. muta* venom contains several PLA_2_ isoenzymes (some are basic and others are acidic) that are responsible for hemolytic activity [15,16,42]. At any or higher concentrations evaluated (up to 500 μg/mL), FHS-3 did not induced hemolysis or proteolysis.

### 2.3. Neutralization of Hemorrhage and Myotoxicity

**Figure 3:**
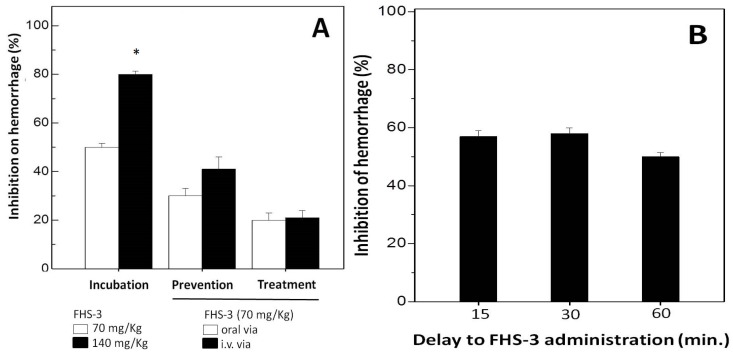
(**A**) For Incubation group, *L. muta* (7 μg/g) venom was incubated with FHS-3 (70 mg/kg or 140 mg/kg) for 30 min, then, mixture was injected s.c.; for Prevention group, FHS-3 (70 mg/kg) was given orally or intravenously, and 15 min later, *L. muta* venom (7 μg/g) was injected s.c.; and for Treatment group, *L. muta* (7 μg/g) venom was injected s.c., and 15 min later, FHS-3 (70 mg/kg) was given orally or intravenously; (**B**) *L. muta* venom (7 μg/g) was injected s.c., and 15 min, 30 min or 60 min after, FHS-3 (70 mg/kg) was injected s.c., and hemorrhage was analyzed. Data are expressed as means SEM of three individual experiments (*n* = 5). * Significance level (*p* < 0.05) when compared to white column.

Hemorrhage caused by SVMPs are due to the degradation of components of the basement membrane (BM), e.g., collagen type IV and laminin and surrounding extra cellular matrix (ECM) which provide mechanical stability to capillaries [43]. SVMPs are zinc-dependent endopeptidases, and are one of the most toxic components of snake venom [43]. This metal ion appears to play a key role in the catalysis as well as to offer structural stability for these enzymes. Injection of *L. muta* venom (7 μg/g) induced a hemorrhage halo in mice of 20 mm, which corresponds to two MHD. Then, the effect of FHS-3 on hemorrhagic activity of such *L. muta* venom concentration was evaluated using the incubation (Figure 3A, group 1), prevention (Figure 3A, group 2) or treatment (Figure 3A, group 3) protocols. As shown (Figure 3A, group 1), when FHS-3 (70 μg/g, white column or 140 μg/g, black column) was incubated with *L. muta* venom, inhibition of hemorrhage was 50% and 80%, respectively. If FHS-3 (70 μg/g) was given orally before (group 2, white column) or after (group 3, white column) injection i.d. of *L. muta* venom, a 30% and 20%-inhibition was achieved, respectively. Moreover, when FHS-3 (70 μg/g) was injected i.v. before *L. muta* venom, the percentage of inhibition has reached 40% (group 2, black column), but when injected after *L. muta* venom (group 3, black column), the inhibitory percentage dropped to half (20%). As another proposal of a treatment for snakebite, *L. muta* venom was injected i.d. into mice, and 15, 30 or 60 min after, FHS-3 was injected i.d. at the same injection site. As seen in Figure 3B, FHS-3 protected mice from hemorrhage by *ca.* 55%, at any period of time evaluated.

**Figure 4:**
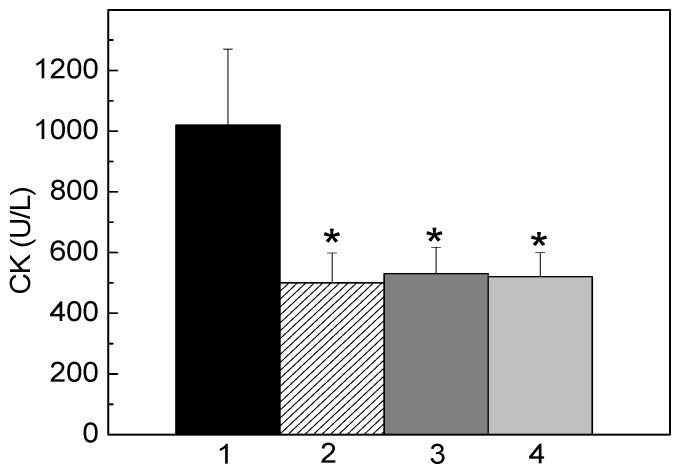
*L. muta* venom (5 μg/g) was injected intramuscularly into mice, and 15 min later, saline (column 1) and FHS-3 (150 μg/g) was injected at the same site of injection of *L. muta* venom (column 2), orally (column 3) or intravenously (column 4). Then, myotoxicity was determined, as described. Data are expressed as means SEM of three individual experiments (*n* = 4). * Significance level (*p* < 0.05) when compared to column 1 (control, *L. muta* venom with saline).

Moreover, when FHS-3 (200 μg/mL) was co-incubated with venom, *L. muta* (5 μg/mL)-induced myotoxic activity was fully inhibited. So, it was interesting to mimic an *in vivo* situation for such activity as well. In this way, *L. muta* venom was firstly injected i.m. into mice, and 15 min later, saline was injected i.m. (Figure 4, column 1) or FHS-3 (150 μg/g) at the site of the injection of *L. muta* venom (Figure 4, column 2), orally (Figure 4, column 3) or intravenously (Figure 4, column 4). As seen in Figure 4, FHS-3 inhibited myotoxicity, regardless the route of administration. In general, as observed in all protocols, FHS-3 inhibited hemorrhage (Figure 3) and myotoxicity (Figure 4) of *L. muta* venom; and such results may lead us to speculate the use of FHS-3 in order to improve antivenom therapy, to prevent or to diminish local necrosis effects that cause severe disabilities or disfigurement and mortality. However, the inhibitory mechanism of FHS-3 on hemorrhage or myotoxicity may be through chelating Zn^2+^ or Ca^2+^ interacting with specific domains to inactivate the SVMPs or PLA_2_ enzymes, respectively, preventing the induction of hemorrhage or tissue necrosis.

### 2.4. Neutralization of Edema

**Figure 5:**
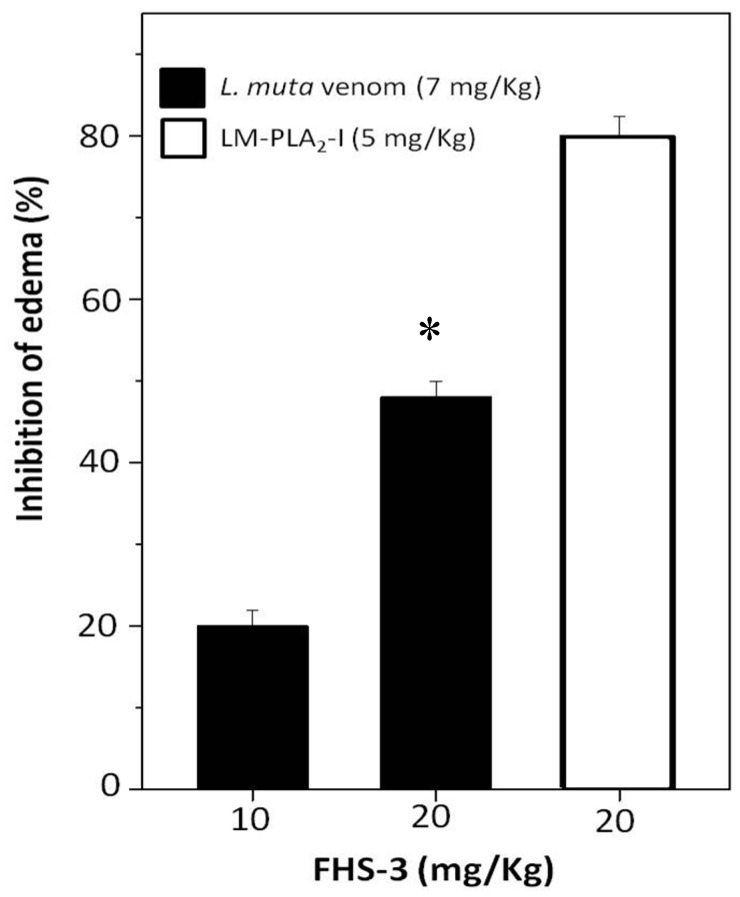
*L. muta* venom (7 μg/g) was mixed with FHS-3 (10 μg/g or 20 μg/g). LM-PLA_2_-I (5 μg/g) was mixed with FHS-3 (20 μg/g). Then, mixtures were incubated for 30 min at 25 °C, injected into mice and edema analyzed, as described in Methods. Data are expressed as means SEM of three individual experiments (*n* = 5). * Significance level (*p* < 0.05) when compared to *L. muta* venom mixed with FHS (10 mg/kg).

Edema is another important pathophysiological effect of snake bites, and PLA_2_ enzymes, which enzymatic activity is Ca^2+^-dependent are involved as well. After sub plantar injection of *L. muta* (7 mg/kg) venom or LM-PLA_2_-I (5 mg/kg) mixed with saline into the paw of mice, an increase of 40% was observed in their volume, and such increase was considered as 100% of edematogenic activity. When such *L. muta* venom dose was incubated with FHS-3 at 10 mg/kg or 20 mg/kg, edema was reduced by 20% and 50%, respectively (Figure 5). Moreover, FHS-3 at 20 mg/kg inhibited LM-PLA_2_-I (5 mg/kg)-induced edema by 80% (Figure 5). So, FHS-3 partially inhibited edema caused by injection of *L. muta* venom as well as the edema of induced by LM-PLA_2_-I, previously isolated [15]. Neither FHS-3 nor saline induced edema. Taken together, FHS-3 inhibited the main toxic activities of *L. muta* venom, but its mechanism of action is under investigation, and the binding of FHS-3 to divalent metals, as Ca^2+^ or Zn^2+^ should be taken into consideration, since its structure has negative charges, and most of the enzymes of *L. muta* venom require such metals to display toxic effects. Thus, neutralization of edema-inducing activity of *L. muta* venom suggests the inhibition of inflammatory reactions as well as myonecrosis and dermonecrosis, are most likely due to PLA_2_ activity of the venom. Although only few studies using sulfated polysaccharides from seaweeds describe these natural compounds as antivenom agents [24,25], some anionic polysaccharides from animal sources as heparin and heparan sulfate have been shown to interact *in vitro* and *in vivo* with different types of PLA_2_s, inhibiting their enzymatic toxic effects [44]. The mechanism of snake venom toxins neutralization by glycosaminoglycans of the heparin/heparan sulfate family is based on the formation of multimolecular complexes, which are stabilized by electrostatic interactions between basic amino acid residues of the PLA_2_s and negative charges proportioned by these sulfated polysaccharides. Additionally, the presence of the 2-*O*-sulfates was described as more prominent role than 6-*O*-sulfates for the interaction heparin–PLA_2_s binding surface [45]. For the biological activities, not only the degree of sulfation, but the sulfate groups positioning is a determinant factor. Sulfated galactan enriched with diads constituted by 3-linked β-d-galactose 2-sulfate and 4-linked α-l-galactose 6-sulfate obtained from *Acanthophora spicifera* showed high antiherpetic activity. This antiviral effect was attributed to an interaction between this sulfated galactan with the glycoprotein C of HSV-1. Due to the pattern of sulfation the sulfated galactan could present a similar conformation to that minimal sequence of the heparan sulfate (HS), inhibiting the interaction of the viral glycoprotein C and HS on the cell surface [37]. The galactan FHS-3 presents 19.2% of sulfate groups, that are located principally at C-2 of the 3-linked β-d-galactosyl units (A units). Additionally, minor quantities of these negative charged groups are present in C-4 and C-6 of the A units. In the B units, sulfate groups occupy principally C-6 of the 4-linked α-l-galactosyl units and C-2 of the 4-linked 3,6-anhydro-α-l-galactosyl units. Minor amounts of sulfate groups are present in C-2 and C-3 of the 4-linked α-l-galactosyl units [31]. In this way, some inhibitory effects of the sulfated galactan FHS-3, such as hemolysis and edema, could be attributed at least in part to their pattern of sulfation. Similarly, as described for the heparin/heparan sulfate family, the sulfate position at C-2 of the 3-linked β-d-galactosyl units could be an important factor for the inhibitory effect of the sulfated galactan FHS-3 against *L. muta* venomand LM-PLA_2_-I.

FHS-3 inhibited the coagulant (Figure 1), proteolytic/hemolysis (Figure 2) and hemorrhagic (Figure 3) and myotoxic (Figure 4) activities of the venom of *L. muta* that are caused by serine, metalloproteases and/or phospholipases A_2_. These enzymes of *Lachesis* and *Bothrops* genera act in the local tissue damage, being associated with deaths and disabilities in envenomation by snakes. Since antivenom therapy does not block such tissue damage, leading victims to lose their members, alternative treatments for snake bites are relevant. It is worth highlighting that, in the literature there are many articles showing antivenom effect of molecules or crude extracts from natural sources, just when such bioproducts were preincubated with snake venoms. However, in this work, different approaches were tested to analyze the antivenom property of FHS-3, and protocols mimicking real situations of envenomation were employed.

## 3. Experimental Section

### 3.1. Venom and Animals

*L. muta* snake venom was kindly supplied by Fundação Ezequiel Dias (FUNED), Belo Horizonte, Minas Gerais state, Brazil, vacuum dried and stored at −20 °C until use. A phospholipase A2 (LM-PLA_2_-I) was isolated from *L. muta*, as previously described [15]. Balb/c mice (18–20 g) were obtained from the Núcleo de Animais de Laboratório (NAL) of the Federal Fluminense University (UFF), and were housed under constant temperature (24 ± 1 °C) and light conditions. Experiments performed were approved by the UFF Institutional Committee for Ethics in Animal Experimentation (protocol number 25) and were in accordance with the guidelines of the Brazilian Committee for Animal Experimentation (COBEA).

### 3.2. Algal Collection and Isolation of the Sulfated Galactan FHS-3

Specimens of the red seaweed *P. flagellifera* (J. Agardh) K. W. Nam were collected at the northeast coast of Brazil (Cabo, Pernambuco State). An exemplar specimen was deposited at the herbarium of the Department of Botany, Federal University of Paraná (Curitiba, Brazil) with the identification code UPCB 55549. The algal material was cleaned to remove contaminants, washed with tap water, sun-dried and milled. Whole thalli were used for the polysaccharide extractions. The sulfated galactan FHS-3 was obtained as previously described [30,31]. Briefly, the dried and milled seaweed *P. flagellifera* was successively extracted (3×) with water at 25 °C, and the extracts were pooled giving rise to the FC fraction (13% yield, based on dried and milled seaweed). The algal residue was then extracted (3×) with water at 80 °C, and the extracts were combined yielding the FH fraction (15% yield, based on dried and milled seaweed). The hot aqueous extract was treated with 2 M KCl, and after centrifugation the supernatant originated the KCl-soluble fraction FHS (69.0% yield, based on FH fraction). FHS was fractionated on a DEAE-Sephacel column that was sequentially eluted with water and NaCl solutions of increasing concentrations (0.1–4.0 M) to give five subfractions (from FHS-1 to FHS-5). The galactan FHS-3 (35.0% yield, based on FHS fraction) was eluted with 0.6 M NaCl.

The sulfated galactan FHS-3 (10 mg) was resuspended in 1 mL of saline (0.15 M NaCl), aliquoted and kept at −20 °C until assays were carried out. 

### 3.3. Antihemolytic Activity

The degree of hemolysis caused by the venom of *L. muta* was determined by the indirect hemolytic test using human erythrocytes and hen’s egg yolk emulsion as substrate [15,16]. The concentration of *L. muta* venom that produced 100% of hemolysis was denoted as Minimum Indirect Hemolytic Concentration (MIHC). The hemolytic inhibitory effect of the sulfated galactan was determined by incubating one MIHC of *L. muta* venom with FHS-3 at different concentrations (180 and 360 μg/mL) for 30 min 25 °C. Then, the hemolytic activity was evaluated. Control experiments were performed by adding FHS-3 or saline, instead of venom.

### 3.4. Antiproteolytic Activity

Proteolytic activity of *L. muta* venom was determined using azocasein as substrate (0.2% *w*/*v*, in 20 mM Tris-HCl, 8 mM CaCl_2_, pH 8.8), with minor modification [46]. The concentration of *L. muta* venom that was able to produce a variation of about 0.2 at A 420 nm was defined as the Effective Concentration (EC). The proteolytic inhibitory effect of the sulfated galactan was performed by incubating one EC of the *L. muta* venom with FHS-3 (180 and 360 μg/mL) for 30 min 25 °C, and then proteolytic activity was determined. Control experiments were performed by adding FHS-3 or saline, instead of venom.

### 3.5. Anticoagulant Activity

The coagulation activity of *L. muta* venom was determined using a digital Amelung coagulometer, model KC4A (Labcon, Germany). Plasma was kindly obtained from healthy volunteers from a local blood bank of the Hospital Universitário Antônio Pedro of the Federal Fluminense University. *L. muta* venom (50 μL) at different concentrations (2–60 μg/mL) were mixed with 200 μL of a pool of human citrated plasma diluted in saline (1:1), and the concentration of venom that clots plasma in 60 s was called Minimum Coagulant Dose (MCD). To evaluate the effect of the sulfated galactan, one MCD of *L. muta* venom was incubated with FHS-3 at different concentrations (100, 200 and 500 μg/mL) for 30 min 25 °C, and then the mixture was added to plasma and clotting time recorded. Control experiments were performed by adding FHS-3 or saline, instead of venom.

### 3.6. Antihemorrhagic Activity

Hemorrhagic lesion produced by *L. muta* venom was quantified using a procedure described by Kondo [47], with modifications. Briefly, *L. muta* (100 μL) venom was injected subcutaneously (s.c.) into the abdominal skin of mice, and two hours later, the animals were euthanized by decapitation, abdominal skin removed, stretched, and inspected for visual changes in the internal aspect in order to localize hemorrhagic spots. The amount of *L. muta* venom (μg/20 g mouse) that was able to produce a hemorrhagic halo of 10 mm was defined as Minimum Hemorrhagic Dose (MHD). The inhibitory effect of FHS-3 sulfated galactan was performed using two MHD through different protocols, called (1) incubation, where FHS-3 (70 and 140 μg/mouse) was incubated with *L. muta* venom for 30 min at 25 °C, then mixture (100 μL) was injected s.c. into mice; (2) prevention, where (2a) FHS-3 (100 μL) was injected s.c. and 15 min later, *L. muta* venom (100 μL) was injected s.c. at the same site where FHS-3 has been injected; (2b) FHS-3 (100 μL) was given orally or (2c) intravenously (i.v.), and 15 min later, *L. muta* venom (100 μL) was injected s.c.; and (3) treatment, in which *L. muta* venom (100 μL) was injected s.c.; and (3a) 15 min, (3b) 30 min or (3c) 60 min later, FHS-3 (100 μL) was injected s.c. at the same site of venom injection. Hemorrhagic activity was expressed as the mean diameter (in millimeter) of the hemorrhagic halo induced by *L. muta* venom in the absence and presence of the FHS-3. Negative control was performed by injecting saline or FHS-3, instead of venom.

### 3.7. Antiedematogenic Activity

Edema-inducing activity of *L. muta* venom or LM-PLA_2_-I was determined in accordance to [48], with modifications. Groups of five mice received s.c. a single sub plantar injection of 50 μL of either *L. muta* venom (7 μg/g) or LM-PLA_2_-I (5 μg/g) in the right paw, while the left paw received 50 μL of saline. One hour after injection, edema was evaluated and expressed as the percentage of increase in the weight of the right foot pad compared to the left one. The effect of the sulfated galactan on edema was investigated by incubating FHS-3 (10 and 20 μg/g) with *L. muta* venom for 30 min 25 °C, and then the mixture was injected s.c. into mice (right foot pad) and edema was measured.

### 3.8. Antimyotoxic Activity

*L. muta* venom or saline was injected (50 μL) intramuscularly (i.m.) into mice under the right tibial anterior muscles, so that the injected site was positioned just over the *Extensor Digitorius Longus* (EDL) muscle [49]. Blood samples were collected previously to and two hours after injection, the serum was separated by centrifugation and, if necessary it was stored at 4 °C for subsequent determination of the plasma CK activity, determined by using a diagnostic kit (Sigma Chemical Co., St. Louis, MO, USA). The rate of CK release from the isolated muscles was expressed as an increased of the CK release compared to control values. CK activity was expressed as International Units, where 1 U is the enzyme amount that catalyzes the transformation of 1 μmol of substrate at 25 °C. The rate of CK release from the isolated muscle was expressed as enzyme units released into the medium per gram per hour of collection (U/g·h^−1^), as previously reported [50]. Myotoxicity was expressed as the increase of released CK. The inhibition effect of FHS-3 was performed through two protocols: incubation (1), where *L. muta* venom (5 μg/mL) was incubated with FHS-3 (200 μg/mL) or saline for 30 min at 25 °C, then mixture (50 μL) was injected i.m. into mice, and treatment (2), in which *L. muta* venom (50 μL) was injected i.m., and 15 min later, FHS-3 (50 μL) was injected i.m. at the same site of venom injection (2a), orally (2b) or intravenously (2c). In parallel, negative control was performed by injecting saline, instead of FHS. Two hours later, myotoxicity was determined, as described.

### 3.9. Statistical Analysis

Results are expressed as means ± SEM obtained with the indicated number of animals or experiments performed. The statistical significance of differences among experimental groups was evaluated using the Student’s *t*-test. Significance was taken as *p* < 0.05.

## 4. Conclusions

A sulfated galactan isolated from the red marine alga *P. flagellifera* had antivenom potential against the toxic effects of *L. muta* venom. It is worth highlighting that the protocols tested were similar to real snake bite situations. However, additional data would be necessary to understand the inhibitory mechanism of FHS-3. For a more efficient treatment of local venom toxicity, a combination of antivenom molecules would be a logical progression from this work, since snake venoms are composed of several toxins with multiple activities. 
